# The 2020 elephant die-off in Botswana

**DOI:** 10.7717/peerj.10686

**Published:** 2021-01-11

**Authors:** Rudi J. van Aarde, Stuart L. Pimm, Robert Guldemond, Ryan Huang, Celesté Maré

**Affiliations:** 1 Conservation Ecology Research Unit, Department of Zoology and Entomology, University of Pretoria, Hatfield, Gauteng, South Africa; 2 Nicholas School of the Environment, Duke University, Durham, NC, USA

**Keywords:** Botswana, Conservation, Die-off, Dispersal, Elephants, Fences

## Abstract

The cause of deaths of 350 elephants in 2020 in a relatively small unprotected area of northern Botswana is unknown, and may never be known. Media speculations about it ignore ecological realities. Worse, they make conjectures that can be detrimental to wildlife and sometimes discredit conservation incentives. A broader understanding of the ecological and conservation issues speaks to elephant management across the Kavango–Zambezi Transfrontier Conservation Area that extends across Botswana, Namibia, Angola, Zambia, and Zimbabwe. Our communication addresses these. Malicious poisoning and poaching are unlikely to have played a role. Other species were unaffected, and elephant carcases had their tusks intact. Restriction of freshwater supplies that force elephants to use pans as a water source possibly polluted by blue-green algae blooms is a possible cause, but as yet not supported by evidence. No other species were involved. A contagious disease is the more probable one. Fences and a deep channel of water confine these elephants’ dispersal. These factors explain the elephants’ relatively high population growth rate despite a spell of increased poaching during 2014–2018. While the deaths represent only ~2% of the area’s elephants, the additive effects of poaching and stress induced by people protecting their crops cause alarm. Confinement and relatively high densities probably explain why the die-off occurred only here. It suggests a re-alignment or removal of fences that restrict elephant movements and limits year-round access to freshwater.

## Introduction

The sudden die-off of savanna elephants (*Loxodonta africana*) in a small part of northern Botswana may be a matter of conservation concern. It evoked considerable media response and ongoing speculation about its cause of death (see Supplemental Information). Such speculation does not consider possible ecological explanations that could further conservation and the associated management of elephants.

From March to June 2020, routine helicopter-based surveys counted 350 elephant carcases in a fairly small area in the Ngamiland district of northern Botswana. They were between 1 day and 1 month since death (M. Chase, 2020, personal communication). The area where most of these die-offs occurred is administratively known as NG11. It is not protected. Human-elephant conflict has been intense here (Jackson et al., 2008; Songhurst, McCullough & Coulson, 2016; Pozo et al., 2017) and poaching recently became more noticeable (Schlossberg, Chase & Sutcliffe, 2019) (see Supplemental Information for extended references to the elephants of this area). Nonetheless, malicious poisoning and poaching are unlikely to have played a role. Other species were not found dead, and elephant carcases had their tusks intact (Azeem et al., 2020). Here, we consider the ecological factors that likely contributed to the die-off and their conservation implications.

The area is within the largest savanna elephant population in Africa. It is part of the Kavango–Zambezi Transfrontier Conservation Area (KAZA-TFCA) that extends across five countries: Botswana, Namibia, Angola, Zambia, and Zimbabwe (Stoldt et al., 2020). Some 200,000 elephants and 2.5 million people live here across 520,000 km^2^ of sub-Saharan savanna (Stoldt et al., 2020). Thirty-six protected areas of various IUCN categories are parcelled out among communal areas and private land. The KAZA-TFCA harbours about half of the world’s savanna elephants (Robson et al., 2017). It is of global conservation importance but simultaneously, a place where humans and wildlife share space and interact.

As part of a long-running research programme which considers regional elephant populations and their management, we have tracked 10 elephants within NG11 and several hundred elephants in other areas across southern and eastern savanna Africa. We also have compiled data on elephant numbers in this and other areas. In brief, conditions for elephants in NG11 differ from those of non-confined elephants in the surrounding landscapes. We show that NG11’s elephants are isolated by the Okavango River to the south-west and by fences on the other sides. NG11 imprisons the elephants preventing their dispersal when numbers are high or when conditions may become harmful. The population growth rate within NG11 differs from those outside. We argue restricted elephant movements made this sudden die-off much more likely. If a contagious agent were responsible, it would have implications for elephants beyond NG11 and neighbouring NG12 and the consequences of this are important for managing elephant populations across Africa.

At the time of writing in October 2020, the cause of these deaths was no more than media speculation and one scholarly commentary (Azeem et al., 2020). This speculation also holds for the 22 deaths recorded recently in north-west Zimbabwe (N. Greenwood, 2020, personal communication). The apparent lack of fresh samples from carcases and lockdowns of activities to contain the spread of Covid-19 add to the difficulties of establishing the cause of death. We may never have a definitive answer. Instead, the best we can do is to sketch the ecological aspects and setting of the affected area, its elephants, and its surroundings. We aim to inform pragmatic approaches to conservation beyond protected areas. None of the earlier reports on the die-off addresses the broader ecological and conservation implications.

## Methods

### The study area

**Figure 1:**
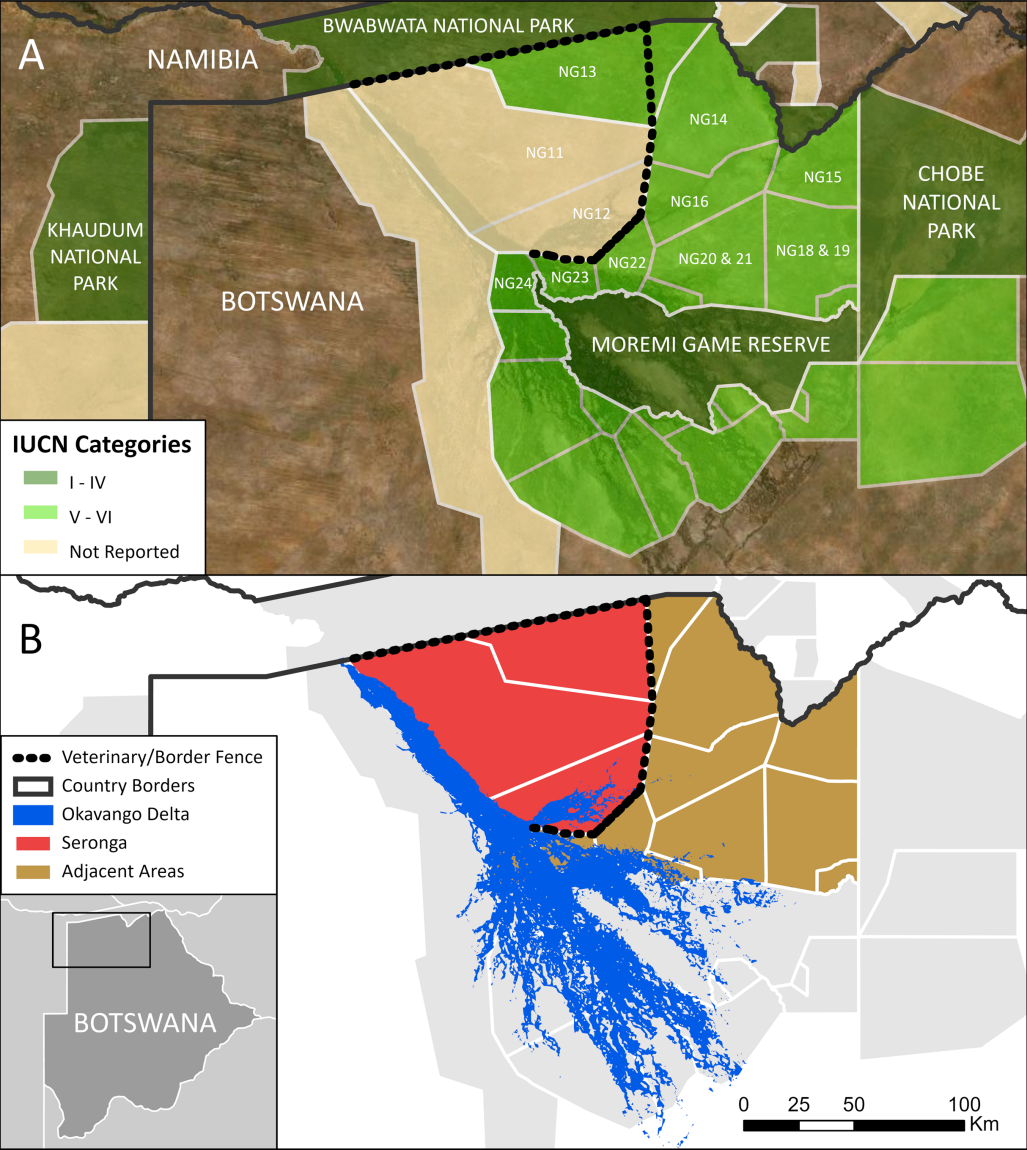
Maps to illustrate administrative and natural boundaries in Ngamiland, northern Botswana. While most of the region has an IUCN status, the NG11 and NG12 administrative blocks have no protected status (A). The Seronga area (NG11, NG12, and a part of NG13) is cut off from the surrounding landscape by either deep water of the Okavango River and Delta (blue) or veterinary or border fences (dashed lines) (B). The shape files for the protected areas were sourced from the World Database on Protected Areas (https://www.protectedplanet.net/en) and for the Okavango Delta from the ESA Climate Change Initiative (Land Cover project 2017).

The die-off occurred in a part of the Ngamiland district of Botswana. We refer to the area of NG11, NG12, and a part of NG13 as the Seronga area (Fig. 1). Some 15,000 elephants live in the Seronga area (Chase et al., 2018). The number of people here increased steadily from about 2,000 in the early 1970s to the latest available census figure of 16,370 in 2011 (Pozo et al., 2017). The Seronga area covers 8,732 km^2^, is roughly triangular, and fences and the deep water of the Okavango River isolate it. To the north, lies the Bwabwata National Park in Namibia along which a fence demarcates the border with Botswana. To the east and south of the region is a veterinary fence, known colloquially as the northern buffalo fence (Fig. 1). It restricts the movement of wildlife and is maintained to prevent the transfer of foot-and-mouth disease from wildlife to domestic animals (Perkins, 2019). The western edge of the region comprises the relatively deep and wide channel of water that forms the Panhandle of the Okavango Delta. The NG11 and NG12 administrative blocks have no protected status. They are designated for subsistence agriculture, though elephants are legally protected (*Wildlife Conservation and National Parks Act, Chapter 38:01, Government of Botswana*). The surrounding areas are protected Wildlife Management Areas with an IUCN V and VI status (Fig. 1). Annual rainfall is relatively low (360–500 mm). Rain falls during the summer (November to April) when daytime temperatures often exceed 35 °C, and when elephants need water and shade for thermoregulation (Mole et al., 2016). Low-growing mopane (*Colophospermum mopane*) dominates the vegetation and provides little shade for elephants. The exception is along the riverine fringes of the Okavango Panhandle, where people live mostly as subsistence farmers in 13 villages (Pozo et al., 2018).

Conflicts between people and elephants in the afflicted area are high in some places and are driven by crop destruction (Jackson et al., 2008; Pozo et al., 2017) (see Supplemental Information). Recently the elephants in the area were poached, resulting in it being designated as one of five poaching hotspots in Botswana (Schlossberg, Chase & Sutcliffe, 2019).

### Monitoring

We fitted tracking devices to 10 adult elephants (seven cows and three bulls) in the Seronga area and tracked their movements from October 2003 to November 2006 (Jackson et al., 2008; Loarie, van Aarde & Pimm, 2009). Africa Wildlife Tracking built the GPS collars, and they provided locations at either 1, 8, 12, or 24 h intervals. We tracked a further 13 adult elephants (eight bulls and five cows) just beyond the boundaries from November 2004 to March 2010. All aspects of the study were subjected to ethical review and were approved by the Animal Ethics Committee of the University of Pretoria (AUCC-040611-013) and were carried out in accordance with international and national guidelines.

We extracted elephant population estimates from previous dry season aerial surveys (see Supplemental Information) to analyse population growth for the Seronga and adjacent areas. Population growth rates are from the slopes of least square regression analyses (weighted with the inverse of variances) fitted to the natural logarithm of elephant numbers over time.

## Results

### Elephant distribution and numbers

**Figure 2:**
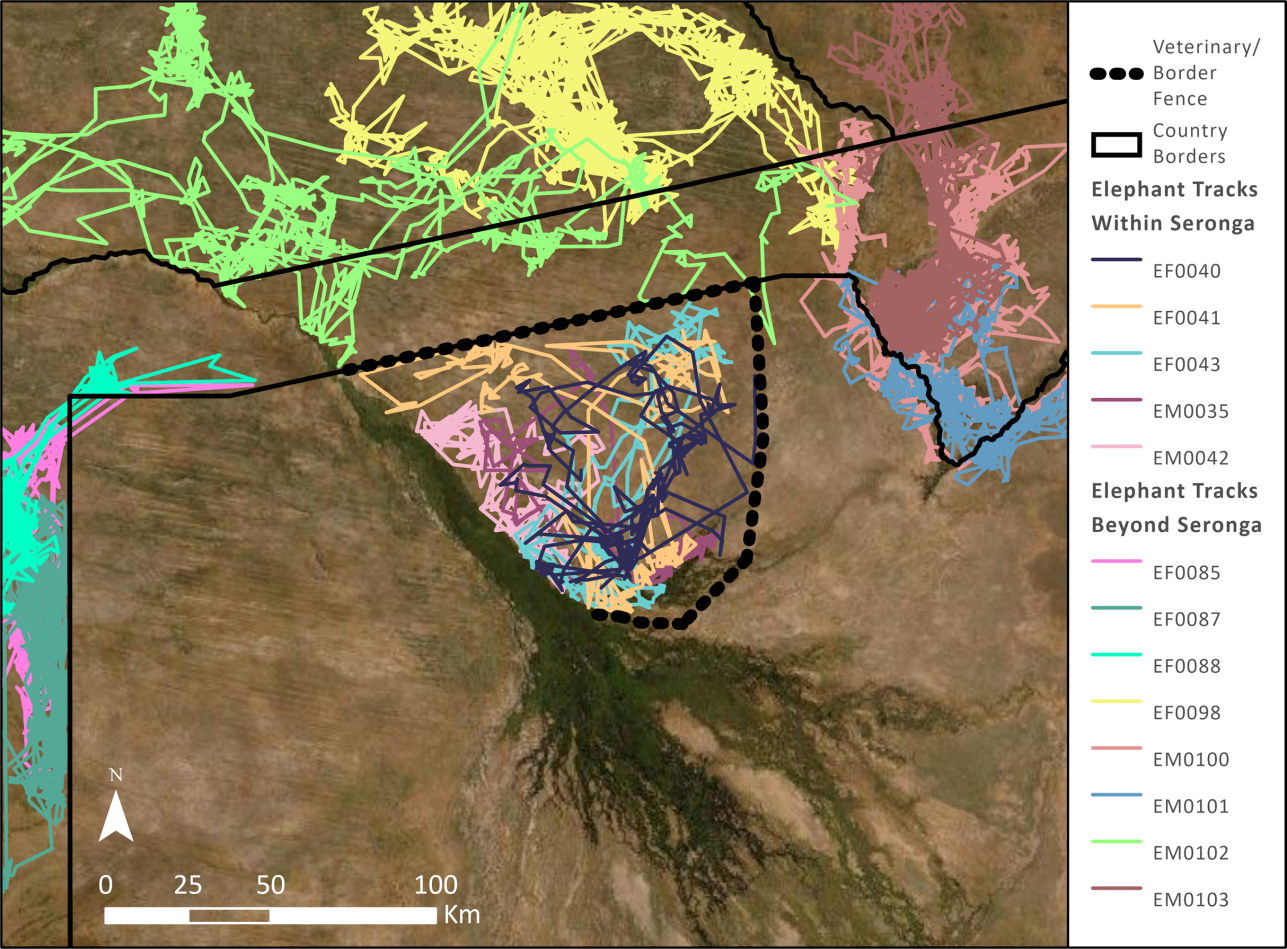
The pathways of five elephants in the Seronga area (NG11, NG12, and NG13) from October 2003 to November 2006 and eight elephants roaming beyond Seronga that we tracked from November 2004 to March 2010. The elephants in Seronga did not cross the veterinary fence (dashed lines) or the Okavango Panhandle, and neither did the elephants beyond the boundaries separating Seronga and the adjacent areas in Botswana, Namibia, Angola, and Zambia. Basemap Source: ESRI, MAXAR.

During 3 years of monitoring, none of the 10 elephants that we tracked in the Seronga area roamed beyond the boundaries formed by the fences or the water channel of the Okavango Panhandle (Fig. 2; for illustrative purposes, we only present the tracks of five elephants.). Similarly, the 13 elephants that we tracked using similar collars beyond NG11, NG12, and NG 13 did not transgress these boundaries (Fig. 2; for illustrative purposes we only present the tracks of eight elephants). The fences and relatively deep water channel of the Okavango River impaired their movement and dispersal. We understand these boundaries are still in place, continuing to impair movement and dispersal. Subsequent studies that tracked 25 collared elephants confirmed our earlier findings (Lindsay et al., 2017; Pozo et al., 2018).

During a dry season aerial survey, about 50% of the elephant observations were within 10 km and >90% within 20 km of the Okavango Panhandle (Jackson et al., 2008). A subsequent wet season aerial survey of the same region revealed <10% of the observations were within 10 km and about 50% within 20 km of the Panhandle. Simply, elephants rely on water in the Okavango Panhandle during the dry season. They move away from people and into the hinterland during the wet season (Jackson et al., 2008). In the dry season, elephants avoided risks by using the night time hours to reach permanent water (Loarie, van Aarde & Pimm, 2009; Ihwagi et al., 2018).

Elephant numbers reported for NG11 and NG12 are much higher at 15,163 ± 1,548 SE (95% CI [12,128–18,198]) than those in the adjacent administrative blocks. The latter range from 150 to 9,000 elephants across NG14 to NG24. These yield an estimated total number of elephants for the adjacent areas collectively at 24,898 ± 1,449 SE (95% CI [22,057–27,739]) (data from Chase et al. (2018) with permission from M. Chase, 2020, personal communication). Elephant density for the Seronga area—2.31 elephants per km^2^—are comparable to the 2.41 elephants per km^2^ collectively for the adjacent areas. The contrast is that these latter elephant populations are not isolated; individuals are free to disperse and have unhindered access to the freshwater of rivers in the region throughout the year (see Fig. 1).

**Figure 3:**
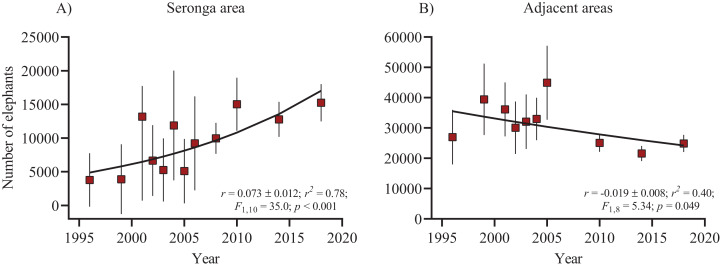
Time series of elephant population estimates from 1996–2018 and exponential fit (weighted with the inverse of variances) in the Seronga area (NG11 and NG12) (A) and the NG14 to NG24 adjacent areas (B). Error bars represent the 95% confidence interval.

Elephants have increased in the Seronga area over the last 23 years (*r* = 0.073 ± 0.012 SE) (Fig. 3A). In contrast, numbers in the adjacent areas have remained stable over the same period (*r* = −0.019 ± 0.008 SE), but fluctuated widely between 1996 and 2018 (Fig. 3B), probably due to movements between the survey areas and years.

## Discussion

In Africa, elephants are intensely poached (Wasser et al., 2015) (see Supplemental Information). Poaching is episodic (Wittemyer et al., 2014; Wasser et al., 2015). It has become one of the most important agents of mortality and drivers of population trends and numbers (Wittemyer et al., 2014; Robson et al., 2017). In some places in sub-Saharan Africa, there are spates of deaths from cyanide poisoning linked with poaching of elephants and other wildlife (Ogada, Botha & Shaw, 2016). There are few published records of die-offs of elephants due to factors other than poaching. We list some of them in our Supplemental Information.

Subsistence farming and other human activities along the Okavango Panhandle restrict the river as a water source for elephants (Jackson et al., 2008; Pozo et al., 2018). From work in Botswana and elsewhere in Africa, elephants tend to avoid areas where people are active (Roever, van Aarde & Chase, 2013; Roever, van Aarde & Leggett, 2013). In the past, elephants in the Seronga area moved across the belt of human activity along the Okavango Panhandle, usually at night when people were inactive, thereby avoiding direct contact and possible harassment (Jackson et al., 2008; Loarie, van Aarde & Pimm, 2009). Pans across the Seronga area filled by rain during the summer are an essential source of water for elephants (Jackson et al., 2008; Loarie, van Aarde & Pimm, 2009), other wildlife, and domestic livestock (~23,000 cattle and ~4,000 goats; Chase et al., 2018). In the dry season, most pans dry out while others may be of low quality and may even become poisonous during blooms of blue-green algae (Azeem et al., 2020).

Should water quality have been low due to rainfall not replenishing the pans in the area, we would have noted an accumulation of carcases of other species (including domestic animals). That might have been similar to the build-up of toxigenic blue-green algae in South Africa’s Kruger National Park in recent years (Bengis et al., 2016). Such algal blooms may have occurred, were reported as a speculation, but not verified. Nor are there reports of signs of malicious poisoning. To explain the elephant deaths, we have to consider other causes, such as disease (Azeem et al., 2020).

Sporadic die-offs caused by naturally occurring diseases play an important role in limiting numbers—as Elton (1931) elegantly voiced nearly a century ago. In particular, elephant populations in sub-Saharan countries, as elsewhere, sporadically die off due to severe and prolonged droughts (Shrader, Pimm & van Aarde, 2010). From a conservation perspective and at the population level, these die-offs may not be of concern and may not call for management interventions. Nonetheless, the additive effects of stress induced by poaching (Gobush, Mutayoba & Wasser, 2008) and people protecting their crops, do cause alarm.

The estimated population growth (7.3%) recorded for the Seronga area is exceptionally high (Pimm, 2008). It falls within the ranges of growth rates for populations confined to small and isolated parks in South Africa (Slotow et al., 2005; Mackey et al., 2006; van Aarde et al., 2008). Two recent case studies of free-ranging elephant populations have similar rates to those in the Seronga area. Foley & Faust (2010) reported a 7.1% growth for the elephant population in Tarangire National Park (Tanzania) that was driven by remarkably high fecundity (age at first calving of 11 years and calving interval at 3.3 years) and low mortality rate of <1% following intense poaching. Elephant movement out of the Masai Mara to the Serengeti increased the population there at 7% (Morrison et al., 2018). However, the increase in elephant numbers in the Seronga region is likely a consequence of a lack of dispersal opportunities. High reproductive output and low background mortality rate in response to ample resource availability may also have contributed to the relatively high growth rate.

Single factors alone may be an insufficient explanation. A recent (2014–2018) spell of poaching lowered numbers and depressed population growth locally over the last 3 years (Schlossberg, Chase & Sutcliffe, 2019). We review some of the consequences in our Supplemental Materials. Elephants respond to poaching by changing their movement patterns (Ihwagi et al., 2018). Poaching induces stress in elephants (Gobush, Mutayoba & Wasser, 2008), which may challenge their immune system and susceptibility to disease. Such susceptibility, the relatively high densities, and the resulting increased contact between individuals could explain the relatively fast spread of a contagious disease that may have been responsible for this die-off in the Seronga area. It would not have had the same impact in the surrounding areas where elephants could move away when harassed.

### The longer-term, wider causes of the elephant die-off and their implications

In brief, we contend that disease is a likely explanation for this die-off but that it begs the broader questions of why here, why then, and what general lessons can we learn? Several forces likely operated in concert to have caused the die-off of elephants in northern Botswana. Vitally, fencing restricted elephant movement, which confined the death-causing agent to the area where the die-off occurred. Impaired dispersal likely caused the high population growth and enhanced local densities of elephants, which could facilitate the spread of a contagious disease. Past incidences of poaching and limitations on access to the Okavango River due to the relatively high number of people and harassment, may have forced elephants to rely on stagnant water sources and cause stress which increased their susceptibility. Those sources may have hosted the agents of their illness, which would have additionally stressed the elephants. Such a complex chain of events consisting of multiple causes makes communication complex and policy actions intricate. We highlight three important aspects in developing policy for elephant management.

First, there is a debate across the wider African conservation landscape about the impact and so desirability of fences (see Supplemental Information). Generally, veterinary fences contribute to the declines of roan antelope (*Hippotragus equinus*), tsessebe (*Damaliscus lunatus*), and sable antelope (*Hippotragus niger*). They have precluded zebra (*Equus quagga*) and wildebeest (*Connochaetes taurinus*) access to water resulting in severe declines in their numbers (Lindsey et al., 2012; Perkins, 2019). These and other studies illustrate the inefficiency and detrimental effects of the veterinary fences and their consequences for elephants, wildlife, people, and landscapes in Botswana and Namibia (Ringrose, Vanderpost & Matheson, 1997; Chase & Griffin, 2009; Darkoh & Mbaiwa, 2009; Perkins, 2019). Specifically, restricting elephant movement and dispersal in the Seronga area adds another case history of the perils of fences to wildlife (Fig. 2). At the same time, we concur with others that fences under conditions different to northern Botswana, as prevails in South Africa, may benefit conservation (Hayward & Kerley, 2009; Pekor et al., 2019).

Second, this episodic die-off poses complex questions about what should be the management response, if any. Its causes were likely a combination of natural factors and human-caused ones. The same applies to the high population growth and densities, which may have made this population susceptible. Calls to control elephant numbers, either because they are perceived as being unnaturally high or because mass die-offs seem unnatural, should ask: what is unnatural? Certainly, not diseases. Elephants are also theoretically capable of high growth rates (Pimm, 2008). Both disease and high densities considered in isolation ignore ecological processes, such as dispersal, that stabilise elephant numbers at the landscape level (van Aarde & Jackson, 2007). What is unnatural about the Seronga population are the barriers that prevent dispersal and year-round access to running (non-stagnant) water.

Third, our results speak to the general issues of understanding the human-wildlife interface at the centre of much current conservation thinking (Western et al., 2020). This discussion sometimes invokes terms of the land-sparing vs land-sharing debate in conservation (Fischer et al., 2014; von Wehrden et al., 2014). Asking which parts of the KAZA-TFCA are ‘spared’ for elephants—such as the national parks (Fig. 1) and which might be ‘shared’ with them (other areas) seems facile given the spread of contagious diseases and barriers to movement and essential resources. In the absence of opportunity to allow for dispersal, such as in the case here, discussion of the re-alignment of the veterinary fences to the benefit of elephants, people, and conservation is a priority. Concerns about barriers to dispersal apply in other African countries, of course.

As popular megafauna, elephants are particularly prone to media excitement and interest. News headlines that posit unverified hypotheses to garner attention may cause confusion and contradictions. These do not benefit conservation. We must not allow our predilections for simple answers to interfere with reasoned analysis and discussing the broader significance.

## Supplemental Information

10.7717/peerj.10686/supp-1 Supplemental Information 1 Supplemental Information

10.7717/peerj.10686/supp-2 Supplemental Information 2 Population size estimates from 1996 to 2018 for elephants in the Seronga region (NG11, NG12, and a part of NG13) and the Adjacent areas in northern Botswana.

## Additional Information and Declarations

### Data Availability

The following information was supplied regarding data availability: The telemetry data of elephants for the region covered in this article are extremely sensitive, due to risk of it being used for illegal activities. Population size estimates from 1996 to 2018 are available in the Supplemental Files.
