# Supplementary material for: The 2020 elephant die-off in Botswana
Source: PeerJ. 2021 Jan 11;9:e10686. doi: 10.7717/peerj.10686 (PMC7808262; doi:10.7717/peerj.10686)
Supplement: Supplemental Information 1 [file peerj-09-10686-s001.pdf]

Supplemental Information

International media cover

A Google search yielded over a 100 media articles on the mass die-off of elephants in Botswana and we list some of them here:

- http://www.elephant-news.com/index.php?id=7412
- https://www.telegraph.co.uk/news/2020/07/01/hundreds-elephants-die-mysteriously-botswana-prompting-fears/
- https://www.thesun.co.uk/news/12105753/deadly-elephant-virus-jump-humans-experts-warn-africa/
- https://www.cbsnews.com/news/elephants-dying-in-africa-botswana-mystery-lab-results-expected-expert-warns-herd-could-be-decimated/
- https://www.livescience.com/elephant-mass-deaths-botswana.html
- https://thehill.com/changing-america/opinion/509867-to-botswana-you-are-the-last-great-hope-for-the-elephant-on-earth
- https://www.insider.com/botswana-death-three-hundred-elephants-is-mystery-after-inconclusive-tests-2020-8
- https://www.businessinsider.com/photos-390-botswana-elephants-found-inexplicably-dead-near-water-2020-7?r=US&IR=T
- https://www.express.co.uk/news/world/1308476/botswana-elephant-deaths-mystery-virus-new-pathogen-okavango-delta-coronavirus
- https://africageographic.com/stories/botswana-elephant-graveyard-mystery-death-toll-rises-to-400/

General references to the importance of poaching

Archie EA, Chiyo PI. 2012. Elephant behaviour and conservation: social relationships, the effects of poaching, and genetic tools for management. *Molecular Ecology* **21**:765-778 https://doi.org/org/10.1111/j.1365-294X.2011.05237.x.

Douglas-Hamilton I, Krink T, Vollrath F. 2005. Movements and corridors of African elephants in relation to protected areas. *Naturwissenschaften* **92**:158–163 https://doi.org/10.1007/s00114-004-0606-9.

Gobush KS, Mutayoba BM, Wasser SK. 2008. Long-term impacts of poaching on relatedness, stress physiology, and reproductive output of adult female African elephant. *Conservation Biology* **22**:1590-1599 https://doi.org/10.1111/j.1523-1739.2008.01035.x.

Goldenberg SZ, Douglas-Hamilton I, Wittemyer G. 2018. Inter-generational change in African elephant range use is associated with poaching risk, primary productivity and adult mortality. *Proceedings of the Royal Society London B* **285**:20180286 https://doi.org/10.1098/rspb.2018.0286.

Hunninck L, Ringstad IH, Jackson CR, May R, Fossøy F, Uiseb K, Killian W, Palme R, Røskaft E. 2017. Being stressed outside the park–conservation of African elephants (*Loxodonta africana*) in Namibia. *Conservation Physiology* **5**:cox067 https://doi.org/10.1093/conphys/cox067.

Ihwagi FW, Thouless C, Wang T, Skidmore AK, Omondi P, Douglas-Hamilton I. 2018. Night-day speed ratio of elephants as indicator of poaching levels. *Ecological Indicators* **84**:38–44 https://doi.org/10.1016/j.ecolind.2017.08.039.

Muboko N, Muposhi V, Tarakini T, Gandiwa E, Vengesayi S, Makuwe E. 2014. Cyanide poisoning and African elephant mortality in Hwange National Park, Zimbabwe: a preliminary assessment. *Pachyderm* **55**:92–94.

Robson AS, Trimble MJ, Purdon A, Young-Overton KD, Pimm SL, van Aarde RJ. 2017. Savanna elephant numbers are only a quarter of their expected values. *PLoS ONE* **12(4)**:e0175942 https://doi.org/10.1371/journal.pone.0175942.

Schlossberg S, Chase MJ, Gobush KS, Wasser SM, Lindsay K. 2020. State-space models reveal a continuing elephant poaching problem in most of Africa. *Scientific Reports* **10**:10166 https://doi.org/10.1038/s41598-020-66906-w.

Wasser SK, Torkelson A, Winters M, Horeaux Y, Tucker S, Otiende MY, Sitam FAT, Buckleton J, Weir BS. 2018. Combating transnational organised crime by linking multiple large ivory seizures to the same dealer. *Science Advances* **4**:eaat0625 https://doi.org/10.1126/sciadv.aat0625.

Wittemyer G, Northrup JM, Blanc J, Douglas-Hamilton I, Omondi P, Burnham KP. 2014. Illegal killing for ivory drives global decline in African elephants. *Proceedings of the National Academy Science USA* **111**:13117–13121 www.pnas.org/cgi/doi/10.1073/pnas.1403984111.

Poaching and human-elephant conflict in Botswana

Reference documents illustrating that poaching of elephant and human-elephant conflict has been intense in northern Botswana.

Buchhotlz E, Fitzgerald L, Songhurst A, McCulloch G, Stronza A. 2019a. Overlapping landscape utilization by elephants and people in the Western Okavango panhandle:

implications for conflict and conservation. *Landscape Ecology* **34**:1411–1423 https://doi.org/10.1007/s10980-019-00856-1.

Buchhotlz E, Redmore L, Fitzgerald LA, Stronza A, Songhurst AC, McCulloch G. 2019b. Temporal partitioning and overlapping use of a shared natural resource by people and elephants. *Frontiers in Ecology and Evolution* **7**:117 https://doi.org/10.3389/fevo.2019.00117.

Buchhotlz E, Stronza A, Songhurst A, McCulloch G, Fitzgerald L. 2020. Using landscape connectivity to predict human-wildlife conflict. *Biological Conservation* **248**:108677 https://doi.org/10.1016/j.biocon.2020.108677.

Jackson TP, Mosojane S, Ferreira SM, van Aarde RJ. 2008. Solutions for elephant *Loxodonta africana* crop raiding in northern Botswana: moving away from symptomatic approaches. *Oryx* **42**:83–91 https://doi.org/10.1017/S0030605308001117.

Pozo RA, Coulson T, McCulloch G, Stronza AL, Songhurst AC. 2017. Determining baselines for human-elephant conflict: A matter of time. *PLoS ONE* **12(6)**:e0178840 https://doi.org/10.1371/journal.pone.0178840.

Pozo RA, Cusack JJ, McCulloch G, Stronza A, Songhurst A, Coulson T. 2018. Elephant space-use is not a good predictor of crop-damage. *Biological Conservation* **228**:241–251 https://doi.org/10.1016/j.biocon.2018.10.031.

Roever CL, van Aarde RJ, Chase MJ. 2013a. Incorporating mortality into habitat selection to identify secure and risky habitats for savannah elephants. *Biological Conservation* **164**:98–106 https://doi.org/10.1016/j.biocon.2013.04.006.

Schlossberg S, Chase MJ, Sutcliffe R. 2019. Evidence of a growing elephant poaching problem in Botswana. *Current Biology* **29**:2222–2228 https://doi.org/10.1016/j.cub.2019.05.061.

Songhurst A, Coulson T. 2014. Exploring the effects of spatial autocorrelation when identifying key drivers of wildlife crop-raiding. *Ecology and Evolution* **4**:582–593 https://doi.org/10.1002/ece3.837.

Songhurst A, McCullough G, Coulson T. 2016. Finding pathways to human-elephant coexistence: a risky business. *Oryx* **50**:713-720 https://doi.org/10.1017/S0030605315000344.

Wasser SK, Gobush KS. 2019. Conservation: Monitoring elephant poaching to prevent a population crash. *Current Biology* **29**:R623–R646 https://doi.org/10.1016/j.cub.2019.06.009.

Droughts

Corfield TF. 1973. Elephant mortality in Tsavo National Park, Kenya. *African Journal of Ecology* **11**:339–368 https://doi.org/10.1111j.1365-2028.tb00098.x.

Douglas-Hamilton I. 1987. African elephants: population trends and their causes. *Oryx* **21**:11–24 https://doi.org/10.1017/S0030605300020433.

Dudley JP, Craig GC, Gibson DStC, Haynes G, Klimowicz J. 2001. Drought mortality of bush elephants in Hwange National Park, Zimbabwe. *African Journal of Ecology* **39**:187–194 https://doi.org/10.1046/j.0141-6707.2000.00297.x.

Walker BH, Emslie RH, Owen-Smith RN, Scholes RJ. 1987. To cull or not to cull: Lessons from a southern African drought. *Journal of Applied Ecology* **24**:381–401 https://doi.org/10.2307/2403882.

Wato YA, Heitkönig IMA, van Wieren SE, Wahungu G, Prins HHT, van Langevelde F. 2016. Prolonged drought results in starvation of African elephant (*Loxodonta africana*). *Biological Conservation* **203**:89–96 https://doi.org/10.1016/j.biocon.2016.09.007.

The role of diseases

There are few published records of die-offs of elephants due to factors other than poaching. These often are due to viral and bacterial diseases (*Grobler et al., 1995*; *Azeem et al., 2020*). For instance, in South Africa's Kruger National Park, the encephalomyocarditis (EMC) virus (EMCV-1; *van Sandwyk et al., 2013*) caused the death of at least 64 elephants during 1993-1994. Apparently, it coincided with an outbreak in rodent numbers (*Grobler et al., 1995*). High rodent numbers in an area where elephant densities are high, may provide the ingredients for such a die-off. As yet, we have no evidence of such an outbreak in the Seronga area. We can posit other infectious candidates such as the West Nile and Shuni viruses (*Azeem et al., 2020*), elephant endotheliotropic herpesvirus (EEHV) which is an infectious type of herpes virus found in elephants (*Richman et al., 1999*), hepatitis (*Basson et al., 1971*; *McCully et al., 1971*), or an unlikely outbreak of tuberculosis (TB) due to slow disease progression and/or chronic infection (*Obanda et al., 2013*; ADS Bastos, 2020, pers. comm.).

Anthrax caused by *Bacillus anthracis* is an indigenous and endemic disease in sub-Saharan Africa (*Hugh-Jones & de Vos, 2002*) that we cannot rule out as a cause of these die-offs. In Etosha National Park, frequent outbreaks of anthrax apparently steer the demography of the elephant population (*Lindeque, 1988*). Outbreaks of anthrax mainly affect elephants during the end of the dry season (*Lindeque & Turnbull, 1994*). During the 2019 drought, 100 elephants died of anthrax in an area 150 km south of the present area (https://africanelephantjournal.com/). In the same year and at the end of the dry season, media reports indicated that the drought caused the death of 200 elephants in Hwange National Park

(Zimbabwe) along a part of its eastern border with Botswana. At least another 100 elephants died from anthrax and at the end of the dry season in Botswana's Chobe National Park. However, in all cases other species were affected as would be anticipated for a multi-species pathogen (ADS Bastos, 2020, pers. comm.)

Azeem S, Bengis R, van Aarde R, Bastos ADS. 2020. Mass die-off of African elephants in Botswana: pathogen, poison or a perfect storm? *African Journal Wildlife of Research* **50**:149–156 https://doi.org/10.3957/056.050.0149.

Basson PA, McCully RM, de Vos V, Young E, Kruger SP. 1971. Some parasitic and other natural diseases of the African elephant in the Kruger National Park. *Onderstepoort Journal of Veterinary Research* **38**:239–254.

Grobler DG, Raath JP, Braack LEO, Keet DF, Gerdes GH, Barnard BJH, Kriek NPJ, Jardine J, Swanepoel R. 1995. An outbreak of encephalomyocarditis-virus infection in free-ranging African elephants in the Kruger National Park. *Onderstepoort Journal of Veterinary Research* **62**:97–108.

Hugh-Jones ME, de Vos V. 2002. Anthrax and wildlife. *Revue Scientific et Technique (International Office of Epizootics)* **21**:359-383 https://doi.org/10.2050/rst.21.2.1336.

Lindeque M. 1988. Population dynamics of elephants in Etosha National Park, S.W.A/Namibia. PhD dissertation, University of Stellenbosch.

Lindeque PM, Turnbull PCB. 1994. Ecology and epidemiology of anthrax in the Etosha National Park, Namibia. *Onderstepoort Journal of Veterinary Research* **61**:71–83.

McCully RM, Basson PA, Pienaar JG, Erasmus BJ, Young E. 1971. Herpes nodules in the lung of the African elephant [*Loxodonta africana* (Blumenbach, 1797)]. *Onderstepoort Journal of Veterinary Research* **38**:225–236.

Obanda V, Poghon J, Yongo M, Mulei I, Ngotho M, Waititu K, Makumi J, Gakuya F, Omondi P, Sorigeur RC, Alasaad S. 2013. First reported case of fatal tuberculosis in a wild African elephant with past human-wildlife contact. *Epidemiology and Infection* **141**:1476–1480 https://doi.org/10.1017/S0950268813000022.

Richman LK, Montali RJ, Garber RL, Kennedy MA, Lehnhardt J, Hildebrandt T, Schmitt D, Hardy T, Alcendor DJ, Hayward GS. 1999. Novel endotheliotropic herpesviruses fatal for Asian and African elephants. *Science* **283**:1171–1176 https://doi.org/10.1126/science.283.5405.1171.

van Sandwyk JHdT, Bennett NC, Swanepoel R, Bastos ADS. 2013. Retrospective genetic characterisation of *Encephalomyocarditis viruses* from African elephant and swine recovers two distinct lineages in South Africa. *Veterinary Microbiology* **162**:23–31 https://doi.org/10.1016/j.vetmic.2012.08.008.

Sources of aerial survey data

We extracted elephant population estimates from aerial surveys (*DWNP 1996, 1999, 2001, 2002, 2003, 2004, 2005; Chase 2011; Chase et al., 2014; Songhurst et al., 2015; Chase et al., 2018*) to analyse population growth for Seronga and the adjacent areas.

Chase M. 2011. Dry season fixed-wing aerial survey of elephants and wildlife in northern Botswana (September-November 2010). Report. Elephants Without Borders, Department of Wildlife and National Parks (Botswana) and the Zoological Society of San Diego.

Chase M, Schlossberg S, Landen K, Sutcliffe R, Seonyatseng E, Keitsile A, Flyman M. 2014. Dry season aerial survey of elephants and wildlife in northern Botswana (July-October 2014). Report. Elephants Without Borders and the Department of Wildlife and National Parks (Botswana).

Chase M, Schlossberg S, Sutcliffe R, Seonyatseng E. 2018. Dry season aerial survey of elephants and wildlife in northern Botswana, July – October 2018. Elephants Without Borders and the Department of Wildlife and National Parks (Botswana).

DWNP. 1996. Aerial census of wildlife and some domestic animals in Botswana. Dry season report. Department of Wildlife and National Parks, Government of Botswana, Gaborone.

DWNP. 1999. Aerial census of wildlife and some domestic animals in Botswana. Dry season report. Department of Wildlife and National Parks, Government of Botswana, Gaborone.

DWNP. 2001. Aerial census of wildlife and some domestic animals in Botswana. Dry season report. Department of Wildlife and National Parks, Government of Botswana, Gaborone.

DWNP. 2002. Aerial census of wildlife and some domestic animals in Botswana. Dry season report. Department of Wildlife and National Parks, Government of Botswana, Gaborone.

DWNP. 2003. Aerial census of wildlife and some domestic animals in Botswana. Dry season report. Department of Wildlife and National Parks, Government of Botswana, Gaborone.

DWNP. 2004. Aerial census of wildlife and some domestic animals in Botswana. Dry season report. Department of Wildlife and National Parks, Government of Botswana, Gaborone.

DWNP. 2005. Aerial census of wildlife and some domestic animals in Botswana. Dry season report. Department of Wildlife and National Parks, Government of Botswana, Gaborone.

Songhurst A, Chase M, Coulson T. 2015. Using simulations of past and present elephant (*Loxodonta africana*) population numbers in the Okavango Delta Panhandle, Botswana to improve future population estimates. *Wetlands Ecology and Management* **23**:583–602 https://doi.org/10.1007/s11273-015-9440-4.

The debate about fences

Creel S, Becker MS, Durant SM, M'Soka J, Matandiko W, Dickman AJ, et al. 2013. Conserving large populations of lions – the argument for fences has holes. *Ecology Letters* **16**:1413-e3 https://doi.org/10.1111/ele.12145.

Jakes AF, Jones PF, Paige LC, Seidler RG, Huijser MP. 2018. A fence run through it: A call for greater attention to the influence of fences on wildlife and ecosystems. *Biological Conservation* **227**:310–318 https://doi.org/10.1016/j.biocon.2018.09.026.

Packer C, Loveridge A, Canney S, Caro T, Garnett ST, Pfeifer M, et al. 2013. Conserving large carnivores: dollars and fences. *Ecology Letters* **16**:635–641 https://doi.org/10.1111/ele.12091.

Riggio J, Caro T, Dollar L, Durant SM, Jacobson AP, Kiffner C, Pimm SL, van Aarde RJ. 2015. Lion populations may be declining in Africa but not as Bauer et al. suggest. *Proceedings of the National Academy of Sciences* **113**:E107–108 www.pnas.org/cgi/doi/10.1073/pnas.1521506113.

Somers MJ, Hayward MW. 2012. *Fencing for conservation. Restriction of evolutionary potential or a riposte to threatening processes?* Springer New York https://doi.org/10.1007/978-1-4614-0902-1.

Woodroffe R, Hedges S, Durant SM. 2014. To fence or not to fence. *Science* **344**:46–48 https://doi.org/10.1126/science.1246251.
